# The prevalence, incidence, and persistence of self-reported visual impairment among Chinese population with diabetes mellitus: evaluation from a nationally representative survey, 2015–2018

**DOI:** 10.3389/fpubh.2023.978457

**Published:** 2023-06-15

**Authors:** Yifan Zhou, Jin Wei, Ning Wang, Yisheng Chen, Cheng Fang, Minwen Zhou, Xinrong Zhou, Jianfeng Luo, Xiaodong Wang, Qing Peng

**Affiliations:** ^1^Department of Ophthalmology, Shanghai Tenth People’s Hospital, School of Medicine, Tongji University, Shanghai, China; ^2^Department of Ophthalmology, Putuo People’s Hospital, Tongji University, Shanghai, China; ^3^Department of Ophthalmology, Shanghai General Hospital, Shanghai, China; ^4^National Clinical Research Center for Eye Diseases, Shanghai, China; ^5^Department of Sports Medicine, Huashan Hospital, Fudan University, Shanghai, China; ^6^Department of Biostatistics, School of Public Health, Fudan University, Shanghai, China; ^7^Key Laboratory of Public Health Safety of Ministry of Education, Fudan University, Shanghai, China; ^8^NHC Key Laboratory of Health Technology Assessment, Fudan University, Shanghai, China; ^9^Shanghai Xin Qi Dian Rehabilitation Hospital, Shanghai, China

**Keywords:** china health and retirement longitudinal survey, diabetes mellitus, vision impairment, prevalence, incidence, persistence

## Abstract

**Aims:**

Our aim was to investigate the prevalence, incidence, and persistence of visual impairment (VI) and their correlates among the Chinese population with diabetes mellitus (DM) over 3 years.

**Materials and methods:**

The China Health and Retirement Longitudinal Survey is the first nationally representative longitudinal survey of the Chinese population. A cross-sectional analysis of prevalent VI in 2015 consisted of 2,173 participants with DM. A longitudinal observation of incident and persistent VI consisted of 1,633 participants from 2015 to 2018. Risk factors of VI were identified via univariate and multivariate logistic regression analyses.

**Results:**

Among our study population with DM, 11.8% reported VI in 2015, 4.5% had persistent VI from 2015 to 2018, and 8.9% developed VI in 2018. Factors identified to be correlated to VI (*p* < 0.05) were older age, being a woman, lower educational attainment, living in a rural area, application of DM medication and non-pharmacological treatment, receiving DM-related tests, use of spectacles, and poorer health status.

**Conclusion:**

This most recent national data provides a baseline for future public health initiatives on VI among the Chinese population with DM. With multiple risk factors identified, these could provide concurrent targets for various public health strategies and interventions with the aim of reducing the burden of VI among the population with DM in China.

## Introduction

Diabetes mellitus (DM) related visual impairment (VI) is currently the world’s leading cause of blindness in the working age and the older population (1), hence, deserving significant attention. The surveillance of VI among the population with DM is essential for evaluating the effectiveness of interventions for VI and other complications of DM (2). The report of the 2016 American National Academies of Science, Engineering, and Medicine calls for a national eye and vision health surveillance system to understand trends, risk factors, and coexistent diseases associated with VI (3). The Centers for Disease Control and Prevention (CDC) of the United States also proposed that data on VI and blindness from national surveys are fundamental components for future national visual surveillance systems (4).

To support such surveillance, national survey data has been analyzed to provide information over the past decades (5, 6). Nationally, representative surveys such as the National Health Interview Survey (NHIS, United States) (2, 7), National Health and Nutrition Examination Survey (NHANES, United States) (8), Health 2000 survey (Finland) (9), Rapid Assessment of Avoidable Blindness and Diabetic Retinopathy Module (RAAB and DRM survey, Hungary) (10), and the Korean National Health and Nutrition Examination Survey (KNHANES, Korea) (11) have been executed to evaluate visual issues, especially VI, among patients with DM in developed countries. To date, there are few nationwide surveys, especially longitudinal studies, evaluating VI among the population with DM from developing countries.

Despite China being the most populous developing country with the most significant number of patients with DM, nationally representative data for VI among the population with DM is yet to be available. In this study, we provide a first for such data using the China Health and Retirement Longitudinal Study (CHARLS), the first nationally representative survey to facilitate health and well-being research among the middle-aged and older Chinese population (persons aged over 45 years) in China. This data provides the prevalence of self-reported VI among the aging population with DM in China. Incident VI (newly developed) and persistent VI were also reported through a longitudinal observation over 3 years. Multiple variables, including socio-demographics, health conditions, and lifestyle-related elements, were analyzed as risk factors.

## Materials and methods

### Participants and public involvement

**Figure 1:**
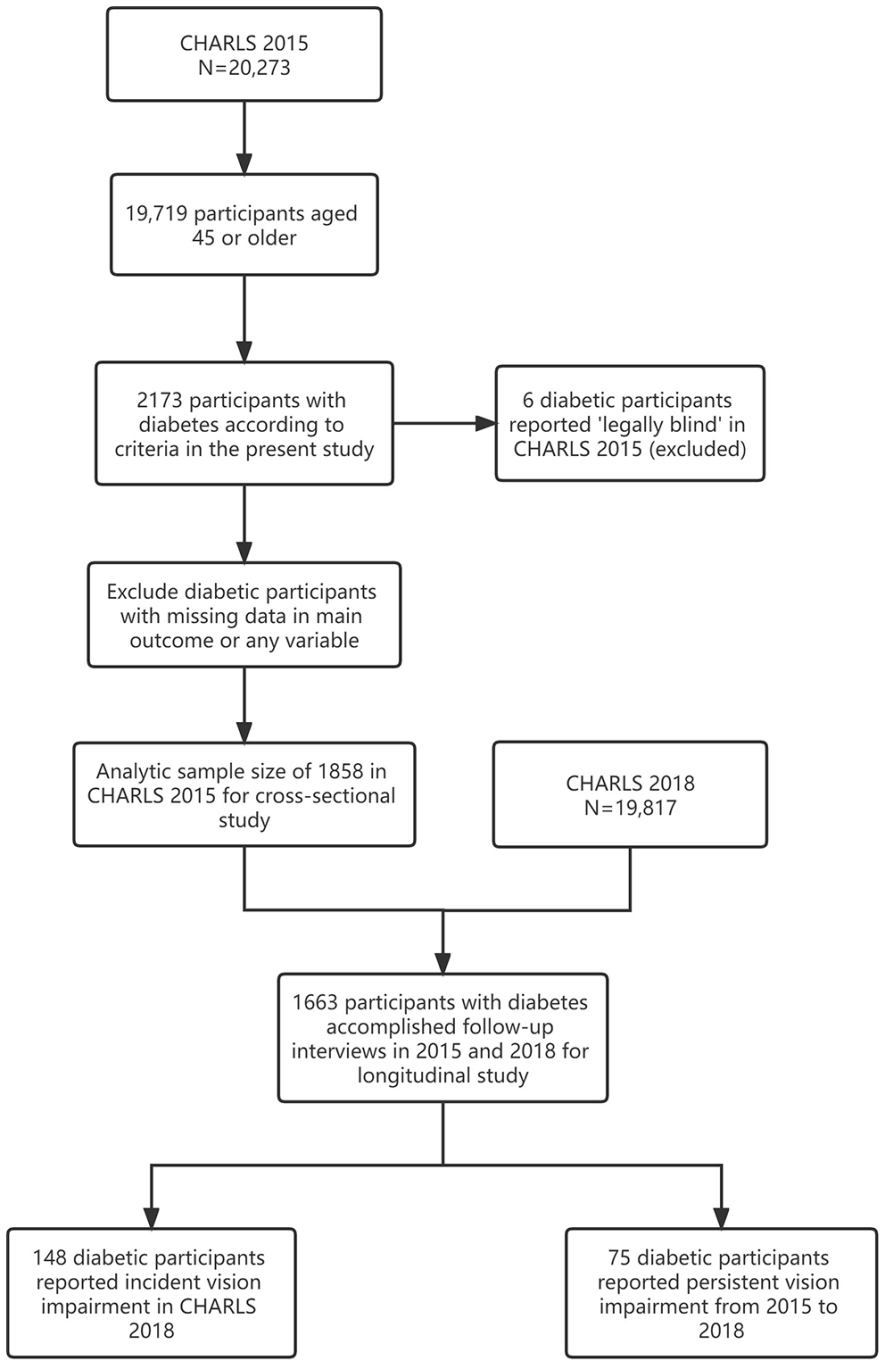
Sample screening of participants with DM in CHARLS 2015 and 2018 for the present study.

We obtained data from the China Health and Retirement Longitudinal Study 2015 (CHARLS 2015, Wave 3, 20,273 participants). Initiated in 2011, CHARLS is the first nationally representative longitudinal survey sampling residents (middle-aged and older population, 45 years old and above) from 450 villages/neighborhoods spanning 150 counties across 28 out of 31 provinces or provincial-level regions in the Chinese mainland. The same study protocol was instituted at all sites. With a response rate exceeding 80%, CHARLS provides the most up-to-date longitudinal data set for assessing the health status and well-being of the middle-aged and older population in China. For the purpose of this study, participants who accomplished the interview in 2015 were adapted for cross-sectional analysis, while those who completed both interviews in 2015 and 2018 were adapted for longitudinal analysis. Participants with missing data were excluded (Figure 1).

### Measures

#### Outcome

The primary outcome in the present study is self-reported VI. In CHARLS, VI included near and distance VI. Near VI and distance VI were assessed by asking participants whether their eyesight was excellent, very good, good, fair, or poor when seeing things up close, like reading ordinary newspaper print (with spectacles or corrective lenses if they usually wear them); or at a distance, like recognizing a friend from across the street (with spectacles or corrective lenses if they usually wear them), respectively. Reports of poor eyesight were classified as near or distant VI. Such categorization of VI assessment was used in previous CHARLS studies (12). Participants reporting both near and distance VI were identified as participants with VI in the current study.

Furthermore, we defined the incidence of VI (newly developed VI) as participants who did not have VI in 2015 but reported VI in the following interview in 2018. Persistent VI was defined as participants who reported VI in both 2015 and 2018.

#### Definition of diabetes mellitus

According to CHARLS 2015, the definition of DM was defined based on both self-reported medical history with previously-diagnosed DM and reference definition (blood test, 1. Fasting plasma glucose level of 126 mg/dL (7.0 mmol/L) or higher; 2. HbA1c concentration of 6.5% or higher) as previously reported (13, 14).

### Independent variables

Multiple factors that could potentially affect VI among patients with DM, reported in previous studies, were adapted into the statistical models in the present study. Also, as many as possible vision-related items in the CHARLS questionnaire were enrolled.

### Socio-demographic characteristics

Age was categorized into four groups (45–54, 55–64, 65–74, and ≥70). Sex was treated as a binary variable: “Male or female” based on the interviewers’ records on the respondent’s gender during a face-to-face interview. Participants who were separated, divorced, widowed, or never married were coded as “living alone,” while those who were married or partnered were coded as “living with a partner.” Living areas referred to urban or rural places where participants lived. Educational level was categorized into five groups: illiterate, less than elementary school, elementary school, middle school, and high school or above.

### Medical condition

Data on medical conditions were collected with the following question: “Have you ever been diagnosed by a doctor as having the following diseases: hypertension, dyslipidemia, diabetes mellitus, cancer, chronic lung diseases, liver diseases, heart disease, stroke, kidney diseases, memory-related diseases, digestive diseases, arthritis, and asthma?” The coexistence of more than two diseases was defined as multi-morbidities (15). Insurance coverage refers to coverage by one or more kinds of health insurance (16).

Specifically, vision-related issues have been included. The use of spectacles is based on the question: “Do you usually wear spectacles or corrective lenses?” The application of DM medication is based on the question: “Are you now taking any medicine or insulin injection to treat or control your DM?” DM management was evaluated by applying non-pharmacological treatment (including weight control, physical exercise, diet, smoking control, and foot care) and DM-related tests (including blood glucose test, urine glucose test, fundus examination, and micro-albuminuria test).

### Statistical methods

The cross-sectional analyses in the present study were performed based on data from CHARLS 2015. Demographic, socioeconomic, and health status and lifestyle-related factors were compared between patients with DM who reported vs. those who denied VI, using analysis of variance, Wilcoxon test, Pearson χ2, or Fisher’s exact test, according to the types and distributions of data. Univariate logistic regressions were conducted to assess the associations between independent variables and VI. Multivariable logistic regressions were performed to determine which variables were independently associated with VI among all independent variables included. Longitudinal observation using univariable and multivariable logistic regressions was conducted to identify risk factors for incident VI (newly developed VI) and persistent VI among patients with DM from 2015 to 2018.

Results were reported as odds ratios (OR) with 95% confidence intervals (95% CI), and *p*-values < 0.05 were regarded as statistically significant in the present study. Statistical analysis was performed using SAS 9.4 statistical software (SAS Institute, Cary, NC, United States).

## Results

### Cross-sectional analyses of data from CHARLS 2015

CHARLS 2015 enrolled 20,273 participants, with 19,719 aged 45 or above from which 2,173 were identified as patients with DM. After excluding participants with missing data, the final analytic sample for the cross-sectional study was 1,858 (Figure 1). The prevalence of VI in our study population was 11.79% (95%CI: 10.35–13.34, Table 1). The prevalence of VI increased with age in both male (2.84%–10.26%, Table 1) and female (10.23%–24.37%, Table 1) populations. Characteristics of the study sample are summarized in Table 2.

**Table 1:** The prevalence of visual impairment among 1,858 participants with DM in CHARLS 2015, stratified by age and gender.

Group	Number	VI	Male			Female					Total	
			Prevalence %	95%CI	Number	VI	Prevalence %	95%CI	Number	VI	Prevalence %	95%CI
45–54	176	5	2.84	0.93–6.50	215	22	10.23	6.52–15.08	391	27	6.90	4.60–9.89
55–64	303	22	7.26	4.61–10.79	393	63	16.03	12.54–20.04	696	85	12.21	9.87–14.87
65–74	253	24	9.49	6.17–13.79	321	46	14.33	10.69–18.65	574	70	12.20	9.63–15.16
75-	78	8	10.26	4.53–19.21	119	29	24.37	16.97–33.09	197	37	18.78	13.58–24.94
Total	810	59	7.28	5.59–9.30	1,048	160	15.27	13.14–17.59	1,858	219	11.79	10.35–13.34

**Table 2:** Characteristics of 1,858 participants with DM in CHARLS 2015.

Variables	Total	Vision impairment	Non-vision impairment	*p* value
Gender				**<0.001**
Male	810 (43.6%)	59 (26.9%)	751 (45.8%)	
Female	1,048 (56.4%)	160 (73.1%)	888 (54.2%)	
Age	62.86 ± 8.99	64.93 ± 8.90	62.59 ± 8.97	**<0.001**
45–54	391 (21.0%)	27 (12.3%)	364 (22.2%)	
55–64	696 (37.5%)	85 (38.8%)	611 (37.3%)	
65–74	574 (30.9%)	70 (32.0%)	504 (30.8%)	
75–	197 (10.6%)	37 (16.9%)	160 (9.8%)	
Marital status				0.3358
Living alone	338 (18.2%)	45 (20.5%)	293 (17.9%)	
Live with partner	1,520 (81.8%)	174 (79.5%)	1,346 (82.1%)	
Education				**<0.001**
Illiterate	503 (27.1%)	96 (43.8%)	407 (24.8%)	
Less than primary school	366 (19.7%)	43 (19.6%)	323 (19.7%)	
Junior School	409 (22.0%)	38 (17.4%)	371 (22.6%)	
Middle school or vocational school	368 (19.8%)	34 (15.5%)	334 (20.4%)	
High school and above	212 (11.4%)	8 (3.7%)	204 (12.4%)	
Living area				**0.0082**
Urban area	390 (21.0%)	31 (14.2%)	359 (21.9%)	
Rural area	1,468 (79.0%)	188 (85.8%)	1,280 (78.1%)	
Use of spectacles				0.2544
Yes	536 (28.8%)	56 (25.6%)	480 (29.3%)	
No	1,322 (71.2%)	163 (74.4%)	1,159 (70.7%)	
DM medication				**<0.001**
Yes	620 (33.4%)	99 (45.2%)	521 (31.8%)	
No	1,238 (66.6%)	120 (54.8%)	1,118 (68.2%)	
DM-related tests				**<0.001**
Yes	711 (38.3%)	108 (49.3%)	603 (36.8%)	
No	1,147 (61.7%)	111 (50.7%)	1,036 (63.2%)	
Diabetic clinic visit				0.5393
Yes	166 (8.9%)	22 (10.0%)	144 (8.8%)	
No	1,692 (91.1%)	197 (90.0%)	1,495 (91.2%)	
Non-pharmacological treatment				**0.0267**
Yes	591 (31.8%)	84 (38.4%)	507 (30.9%)	
No	1,267 (68.2%)	135 (61.6%)	1,132 (69.1%)	
Cataract surgery				0.5656
Yes	64 (3.4%)	9 (4.1%)	55 (3.4%)	
No	1,794 (96.6%)	210 (95.9%)	1,584 (96.6%)	
Multi-morbidities				**<0.001**
Yes	846 (45.5%)	140 (63.9%)	706 (43.1%)	
No	1,012 (54.5%)	79 (36.1%)	933 (56.9%)	
Insurance coverage				0.953
Yes	1,824 (98.2%)	215 (98.2%)	1,609 (98.2%)	
No	33 (1.8%)	4 (1.8%)	29 (1.8%)	

Compared with the non-VI group, there was a significantly larger proportion of women and the aging population in the VI group (all *p* < 0.001; Table 2). Also, VI patients were found to be more likely to live in rural areas, have lower educational attainment, have more DM-related self-managements including the application of DM medication and non-pharmacological treatment, receiving DM-related tests (all *p* < 0.05; Table 2), and have poorer health status (multi-morbidities, *p* < 0.001; Table 2).

According to the univariate logistic regression analysis, factors including age, gender, educational level, living area, multi-morbidities, application of DM medication, non-pharmacological treatment, and receiving DM-related tests were found to be significantly associated with the prevalence of VI among patients with DM (all *p* < 0.05; Table 3). In the multivariate logistic model, only age, gender, educational level, living area, and multi-morbidities remained significantly correlated with VI (all *p* < 0.05; Table 3).

**Table 3:** Logistic regression analyses to identify associated factors of the prevalence of visual impairment in CHARLS 2015.

Variables	Univariate	*p*-value	Multivariate	*p*-value
Gender	Female (reference)			
Male	**0.436 (0.318,0.596)**	**<0.0001**	**0.570 (0.404,0.805)**	**0.0014**
Age	**1.029 (1.013,1.045)**	**0.0003**	**1.027 (1.008,1.045)**	**0.0044**
45–54	Reference			
55–64	**1.875 (1.193,2.947)**	**0.0064**		
65–74	**1.876 (1.180,2.984)**	**0.0079**		
75–	**3.118 (1.835,5.295)**	**<0.0001**		
Marital status	Living alone (reference)			
Live with partner	0.842 (0.593,1.197)	0.3384	1.138 (0.778,1.664)	0.5051
Education	Illiterate (reference)			
Less than primary school	**0.564 (0.383,0.832)**	**0.0039**	0.727 (0.480,1.099)	0.1308
Junior school	**0.435 (0.292,0.650)**	**<0.0001**	**0.560 (0.365,0.858)**	**0.0078**
Middle school or vocational school	**0.432 (0.284,0.655)**	**<0.0001**	0.672 (0.419,1.077)	0.0987
High school and above	**0.166 (0.079,0.349)**	**<0.0001**	**0.267 (0.120,0.591)**	**0.0011**
Living area	Urban area (reference)			
Rural area	**1.702 (1.144,2.533)**	**0.0087**	**1.636 (1.063,2.517)**	**0.0252**
Use of spectacles	No (reference)			
Yes	0.829 (0.601,1.143)	0.2527	0.895 (0.637,1.259)	0.5249
DM medication	No (reference)			
Yes	**1.769 (1.329,2.354)**	**<0.0001**	1.510 (0.954,2.389)	0.0784
DM-related tests	No (reference)			
Yes	**1.670 (1.258,2.217)**	**0.0004**	1.201 (0.741,1.949)	0.4573
Diabetic clinic visit	No (reference)			
Yes	1.159 (0.722,1.859)	0.5414	0.719 (0.425,1.214)	0.2169
Non-pharmacological treatment	No (reference)			
Yes	**1.388 (1.037,1.859)**	**0.0276**	0.972 (0.643,1.469)	0.8929
Cataract surgery	No (reference)			
Yes	1.257 (0.612,2.583)	0.5332	0.807 (0.379,1.719)	0.5785
Multi-morbidities	No (reference)			
Yes	**2.345 (1.750,3.143)**	**<0.0001**	**1.983 (1.441,2.729)**	**<0.0001**
Insurance coverage	No (reference)			
Yes	1.033 (0.360,2.965)	0.9524	1.433 (0.460,4.464)	0.5353

### Longitudinal observation from 2015 to 2018

During the 3 years of longitudinal observation, 1,663 participants with DM accomplished follow-up interviews in 2015 and 2018. From this population, 148 participants without VI in 2015 reported newly developed VI in 2018, and 75 participants reported persistent VI from 2015 to 2018. A total of 1,325 participants who reported good vision from 2015 to 2018 were categorized as the reference group (Figure 1).

Factors including age, gender, educational level, use of spectacles, and multi-morbidities were significantly associated with incident VI according to univariate analysis (all *p* < 0.05; Table 4). However, gender lost its significant correlation with incident VI after being adjusted in the multivariate logistic model (Table 4).

**Table 4:** Logistic regression analyses to identify associated factors of incident visual impairment and persistent visual impairment.

	Incident VI		Persistent VI	
	Univariate	Multivariate	Univariate	Multivariate
Gender	Female (reference)			
Male	**0.637 (0.447,0.908)** ^*^	0.830 (0.559,1.231)	**0.270 (0.149,0.487)** ^***^	**0.360 (0.190,0.683)** ^**^
Age	**1.044 (1.024,1.064)** ^***^	**1.038 (1.015,1.061)** ^*^	**1.032 (1.006,1.059)** ^*^	1.023 (0.992,1.054)
55–64	1.215 (0.716,2.063)		1.326 (0.679,2.588)	
65–74	**2.256 (1.356,3.753)** ^**^		1.108 (0.535,2.298)	
75–	**2.426 (1.265,4.653)** ^*^		**3.241 (1.495,7.028)** ^**^	
Marital status	Living alone (reference)			
Live with partner	0.872 (0.564,1.348)	1.276 (0.799,2.037)	0.750 (0.424,1.327)	1.150 (0.620,2.133)
Education	Illiterate (reference)			
Less than primary school	1.031 (0.664,1.600)	1.125 (0.700,1.808)	**0.390 (0.195,0.782)** ^*^	0.498 (0.237,1.047)
Junior School	0.680 (0.427,1.082)	0.732 (0.442,1.214)	**0.387 (0.201,0.744)** ^**^	0.586 (0.289,1.189)
Middle school or vocational school	**0.311 (0.171,0.565)** ^***^	**0.388 (0.202,0.748)** ^**^	**0.360 (0.184,0.705)** ^**^	0.611 (0.282,1.324)
High school and above	**0.200 (0.084,0.475)** ^***^	**0.228 (0.089,0.583)** ^**^	**0.145 (0.044,0.476)** ^**^	**0.255 (0.070,0.936)** ^*^
Living area	Urban area (reference)			
Rural area	1.543 (0.971,2.452)	1.416 (0.860,2.331)	1.491 (0.793,2.801)	1.681 (0.828,3.412)
Use of spectacle	No (reference)			
	**1.519 (1.068,2.162)** ^*^	**1.621 (1.114,2.359)** ^*^	0.847 (0.497,1.444)	0.999 (0.565,1.767)
DM medication	No (reference)			
	1.223 (0.857,1.746)	0.915 (0.521,1.606)	**2.376 (1.488,3.792)** ^***^	2.040 (0.959,4.338)
DM-related tests	No (reference)			
	1.370 (0.972,1.931)	1.434 (0.792,2.596)	**1.999 (1.253,3.189)** ^**^	1.242 (0.566,2.721)
Diabetic clinic visit	No (reference)			
	1.471 (0.867,2.496)	1.187 (0.647,2.179)	1.269 (0.595,2.707)	0.696 (0.301,1.611)
Non-pharmacological treatment	No (reference)			
	1.078 (0.751,1.547)	0.949 (0.564,1.597)	1.534 (0.955,2.464)	0.833 (0.433,1.600)
Cataract surgery	No (reference)			
	1.184 (0.459,3.057)	0.821 (0.306,2.205)	1.908 (0.663,5.495)	1.125 (0.364,3.475)
Multi-morbidities	No (reference)			
	**1.724 (1.224,2.427)** ^**^	**1.481 (1.015,2.163)** ^*^	**2.775 (1.696,4.540)** ^***^	**2.255 (1.323,3.845)** ^*^
Insurance coverage	No (reference)			
	0.444 (0.059,3.331)	0.657 (0.083,5.200)	2.719 (0.790,9.362)	4.089 (0.987,16.94)

^*^p < 0.05; ^**^p < 0.005; ^***^p < 0.0005.

Factors including age, gender, educational level, multi-morbidities, application of DM medication, and receiving DM-related tests were significantly associated with persistent VI according to the univariate analysis. However, only gender, educational level over the high school, and multi-morbidities remained significant correlates of persistent VI in the multivariate logistic model (all *p* < 0.05; Table 4).

## Discussion

DM is a systemic disease most frequently leading to severe ocular complications and resulting VI in both developed and developing countries (1). For the first time, the general condition including the prevalence, incidence, and persistence of VI among the Chinese population with DM is reported at a national level, using data from a nationally representative and longitudinal survey (CHARLS). We are also the first to identify risk factors of VI from a wide range of elements, including socio-demographic, health status, and lifestyle-related factors.

The prevalence of VI, according to the population-based survey, provides a pivotal epidemiological reference for vision assessment and surveillance. In the present study, according to self-reported visual status, the estimated prevalence of VI among our sample with DM is 11.79%, which is higher than the prevalence of VI in general participants from CHARLS (8%; data not shown in tables). The self-reported visual status could adequately capture the envelope of cumulative VI and blindness (17). However, compared to the physical evaluation of visual acuity, self-reported items may also result in a lower estimation of the prevalence of VI (17). The prevalence of VI in the present study is comparable to those reported in previous region-limited population-based studies on patients with DM in our country: [Dongguan Eye Study: 6.8%–15.7% (15); Shanghai, Xinjing community: 15.7% (18) and 5.96% (19); Jiangsu Diabetic Eye Disease Study, Funing County: 13.47%–23.83% (16); Hong Kong, Integrative Community Health Center: (20) and 11.3% (21)]. However, definition disparities of VI or low vision among all these studies could be noticed. On the other hand, several studies focusing on the general population in China reported a relatively lower prevalence of VI [The China Nine-Province Survey: 3.13%–9.51% (22), Baotou Eye Study: 4.65% (23), Beijing Eye Study: 1.4% (24) and Handan Eye Study: 5.9% (25)]. Despite variations among study designs in previous research, these data could indicate indirectly that patients with DM are more prone to VI than the general population in China to some extent.

### Demographic factors: age and gender

Aging is associated with various decrements in body functions, including VI. The prevalence of VI increases with aging in both the general population and people with DM in China (18–25). Consistently, in the present study, the prevalence of VI increased from 6.90% to 18.78% by aging across different age categories (Table 2). We found that patients with VI are significantly older than those without VI (*p* < 0.001). Also, both univariate and multivariate logistic analyses indicated older age as a risk factor for VI among patients with DM (all *p* < 0.005). Along with age-related visual dysfunction, aging would inevitably correlate with a longer duration of DM. Consequently, aging patients with DM may have a higher probability of suffering from ocular complications, including cataracts, refractive error, and diabetic retinopathy (19–21, 22).

Gender difference in VI has not reached a consensus in our general or diabetic population based on previous regional studies (18–25). Based on this nationally representative survey, we noticed gender differences in VI among our population with DM. The prevalence of VI in the female population is higher than that in the male population across every age category, according to our age-stratified prevalence estimation (Table 2). We found a significantly higher proportion of the female population in the VI group than in the non-VI group (*p* < 0.001). We also noticed that the female population was significantly associated with VI according to univariate and multivariate regression analyses. Such finding is consistent with several national surveys from other countries (26, 27). A series of studies have also indicated that VI or low visual acuity is more prevalent among Chinese women (general population) (28). Studies showed that women might have a longer life expectancy, higher HbA1c level (29), and higher prevalence of DM (30) than men, which may contribute to their higher risk of developing DM-related ocular complications and age-related ocular disorders (22). However, whether DM and gender could synergistically affect VI in the Chinese population with DM is yet to be elucidated and deserves further studies.

### Socio-economic factors: educational level and living area

Educational attainment could be considered a surrogate indicator of socioeconomic status, which impacts people’s access to health services and other social and economic resources. Educational level has been defined as an essential factor that affects VI in the general population in our country (22–25). The Dongguan Eye Study was the first to report a potential association between lower education levels and VI in patients with DM (18). Consistent with their findings, we noticed relatively lower educational attainment among patients reporting VI in the present study. Univariate and multivariate logistic analyses also revealed a significant correlation between insufficient education attainment and VI. Lower educational level is generally associated with fewer physical and ocular examinations (31) and poorer controlled blood glucose levels (31), which lead to worse self-management of DM and prognosis of visual outcomes. Educational level is also closely related to self-consciousness and proper understanding of DM in China (32–34). Thus, particular emphasis should be given to those with lower educational attainment in future national VI prevention programs for the population with DM in our country.

The living area is indispensable in explaining the substantial regional variations in the prevalence of VI across China (22). Compared to most other countries with Health and Retirement Studies, China is much more rural and has more undeveloped areas. Previous population-based studies in our country mainly recruited participants in specific communities or counties. Therefore, the urban/rural comparison of VI prevalence often came with different conclusions (23–25, 35). Based on our nationally representative survey, the geographic distribution of our sample is widely divergent from that in other region-limited population-based studies (Table 1). We provide explicit evidence that living areas could significantly impact VI among our population with DM. Living in a rural area was identified as a risk factor of VI according to both univariate and multivariate logistic models, which indicated that the vision care system in our country, especially in rural China, is still inadequate and ineffective in terms of meeting the needs of vision health care due to the uneven distribution of medical resources (36).

### Longitudinal observation: incident VI and persistent VI

Our study is the first to report a longitudinal observation on VI among a nationally representative sample of the Chinese population with DM. In the present study, 1,663 participants with DM accomplished CHARLS interviews in both 2015 and 2018 from which 148 participants reported newly developed VI, which indicated that the incidence of VI among our sample is 8.90%. A total of 75 participants reported persistent VI from 2015 to 2018, representing 4.51% of our study sample. These data provide essential references to the incidence and persistence of VI among the Chinese population with DM.

Compared to risk factors reported in previous studies with cross-sectional designs, our investigation on risk factors of the incident and persistent VI might provide new insights into correlates of VI in the population with DM. Consistent with our cross-sectional findings, lower educational level and multi-morbidities correlate to incident VI and persistent VI, according to univariate and multivariate logistic analyses (Table 4). Other than these two factors, some interesting findings in our longitudinal study deserve further attention.

First of all, among the population with incident VI, gender difference appeared to be insignificant after applying adjustment in the multivariate logistic model. However, univariate and multivariate logistic analyses among the participants with persistent VI indicated profound gender differences. Such a finding raised our concern: female patients seem more likely to suffer from sustained vision-impaired status after developing VI than male patients. Timely intervention and medical resources should be provided in a manner that focuses concerns on female patients with DM reporting newly developed VI to prevent persistent VI.

Another finding to our interest is that the use of spectacles was associated with incident VI among our sample. An uncorrected refractive error has been noticed as one of the most frequent causes of VI in our population with DM (18, 21). Researchers from Hong Kong reported that nearly 70% of cases of VI could be remedied with spectacle correction (21), which means many patients could choose a relatively safe, economical, and straightforward approach to improve visual acuity. On the other hand, visual aids are not always helpful for people with VI owing to severe ocular complications of DM. More importantly, patients with lower educational levels might incorrectly distinguish between ‘ophthalmologist’ and ‘optometrist’ (37). The probability of misdiagnosing a DM-related ocular disorder by an optometrist is far greater than that by an ophthalmologist (38), which deserves certain attention.

Last but not least, the persistent status of VI may jeopardize both physical and mental status in the middle-aged and older population (39–42). Findings of correlates to persistent VI from the present study provide indications to filter specific patients more prone to persistent VI. Female patients, patients with lower educational attainment (less than high school), and patients with poorer health status (with multi-morbidities) may need timely intervention to terminate persistent VI.

### Strengths and limitations

VI is a major problem worldwide. There has been a consensus in recent years that the establishment of a national eye and visual health surveillance system is essential for future eye care, especially for the populations which are more prone to ocular disorders and resulting VI, such as patients with DM. To establish such a surveillance system, related data from nationally representative surveys have been analyzed in some countries over the past decade. To date, there are few nationwide surveys, especially longitudinal studies, covering VI in the general population and, specifically, population with DM, from developing countries. China, being the most populous developing country, also has the largest number of people with DM. Using the most updated data from the first nationally representative longitudinal survey (2015–2018), this present study is the first to evaluate VI in the Chinese population with DM, with regard to the prevalence, incidence, and persistence of VI and their correlates. Our study provides a baseline for future public health initiatives on VI among the Chinese population with DM. Multiple risk factors were identified as targets for various diverse public health strategies and interventions with the aim of reducing the burden of VI among the population with DM in China. We propose that CHARLS could be appropriately used for monitoring populational VI and as a component of China’s future national vision surveillance system.

Nevertheless, the findings in this report are subjected to at least two limitations. First, a significant concern in any population-based study is nonparticipation. CHARLS 2015 had a reasonable cross-sectional response rate of 82.13% and a panel response rate of 86.46% in 2018. However, differences between participants and non-participants can lead to a selection artifact. Also, the community sample enrollment does not cover those living in an institution (e.g., a nursing home). Second, self-reported visual items and documented visual acuity are both widely accepted assessments of vision status in epidemiological studies. Similar to some well-recognized national surveys like NHIS in the United States and the ELSA in England, CHARLS also adopted self-reported data to assess visual status based on questionnaires. Subjective assessment could further acquire visual complaints like anomalopia or metamorphopsia, which could not be reflected by mere visual acuity assessment. However, it is subjected to reporting bias and prone to conceptual cultural differences. On the other hand, documented visual acuity examination required the participants to go to mobile examination centers (the case in the NHANES, for example), which might have influenced nonparticipation. Patients with severe VI or other conditions may have difficulties traveling to mobile examination centers. Thus, we must realize that disparities in definitions of VI or low vision acuity exist in various studies and, consequently, there should be caveats when interpreting the results.

## Conclusion

Based on the most updated nationally representative and longitudinal data, the present study is the first to introduce the condition of visual impairment, including its prevalence, incidence, persistence, and risk factors among the middle-aged and older Chinese population with DM. CHARLS could be appropriately used to monitor visual impairment and as a component of China’s future national vision surveillance system.

## Data availability statement

Publicly available datasets were analyzed in this study. This data can be found at: the current study is a secondary analysis of public data of CHARLS. The original dataset of CHARLS is accessible on http://charls.pku.edu.cn/.

## Ethics statement

The current study is a secondary analysis of CHARLS data. The conduct of CHARLS was approved by the Biomedical Ethics Review Committee of Peking University (approval number: IRB00001052-11015). The patients/participants provided their written informed consent to participate in this study. Written informed consent was not obtained from the individual(s) for the publication of any potentially identifiable images or data included in this article.

## Author contributions

MZ and JL designed the research. XZ, QP, XW, and JW managed the revision progress. CF and YC collected and analyzed the data. YZ and NW drafted the manuscript. YZ, JW, NW, and YC. All authors contributed to the article and approved the submitted version.
