# Corrigendum: The prevalence, incidence, and persistence of self-reported visual impairment among Chinese population with diabetes mellitus: evaluation from a nationally representative survey, 2015–2018

**DOI:** 10.3389/fpubh.2023.1303526

**Published:** 2023-10-11

**Authors:** Yifan Zhou, Jin Wei, Ning Wang, Yisheng Chen, Cheng Fang, Minwen Zhou, Xinrong Zhou, Jianfeng Luo, Xiaodong Wang, Qing Peng

**Affiliations:** ^1^Department of Ophthalmology, Shanghai Tenth People's Hospital, School of Medicine, Tongji University, Shanghai, China; ^2^Department of Ophthalmology, Putuo People's Hospital, Tongji University, Shanghai, China; ^3^Department of Ophthalmology, Shanghai General Hospital, Shanghai, China; ^4^National Clinical Research Center for Eye Diseases, Shanghai, China; ^5^Department of Sports Medicine, Huashan Hospital, Fudan University, Shanghai, China; ^6^Department of Biostatistics, School of Public Health, Fudan University, Shanghai, China; ^7^Key Laboratory of Public Health Safety of Ministry of Education, Fudan University, Shanghai, China; ^8^NHC Key Laboratory of Health Technology Assessment, Fudan University, Shanghai, China; ^9^Shanghai Xin Qi Dian Rehabilitation Hospital, Shanghai, China

**Keywords:** China health and retirement longitudinal survey, diabetes mellitus, vision impairment, prevalence, incidence, persistence

In the published article, in the **affiliation section**, affiliations 1, 2, 6, 7, 8 and 9 were incorrectly ranked.

The incorrect affiliation section was written as: ^1^Department of Ophthalmology, Putuo People's Hospital, Tongji University, Shanghai, China, ^2^Department of Ophthalmology, Shanghai Tenth People's Hospital, Tongji University, Shanghai, China, ^3^Department of Ophthalmology, Shanghai General Hospital, Shanghai, China, ^4^National Clinical Research Center for Eye Diseases, Shanghai, China, ^5^Department of Sports Medicine, Huashan Hospital, Fudan University, Shanghai, China, ^6^Department of Biostatistics, School of Public Health, Fudan University, Shanghai, China, ^7^Shanghai Xin Qi Dian Rehabilitation Hospital, Shanghai, China, ^8^Key Laboratory of Public Health Safety of Ministry of Education, Fudan University, Shanghai, China, ^9^NHC Key Laboratory of Health Technology Assessment, Fudan University, Shanghai, China.

The correct affiliation ranking has been revised as below:

^1^Department of Ophthalmology, Shanghai Tenth People's Hospital, School of Medicine, Tongji University, Shanghai, China, ^2^Department of Ophthalmology, Putuo People's Hospital, Tongji University, Shanghai, China, ^3^Department of Ophthalmology, Shanghai General Hospital, Shanghai, China, ^4^National Clinical Research Center for Eye Diseases, Shanghai, China, ^5^Department of Sports Medicine, Huashan Hospital, Fudan University, Shanghai, China, ^6^Department of Biostatistics, School of Public Health, Fudan University, Shanghai, China, ^7^Key Laboratory of Public Health Safety of Ministry of Education, Fudan University, Shanghai, China, ^8^NHC Key Laboratory of Health Technology Assessment, Fudan University, Shanghai, China, ^9^Shanghai Xin Qi Dian Rehabilitation Hospital, Shanghai, China.

In the published article, there was an error in the **author list**, and authors Jianfeng Luo and Qing Peng were incorrectly ordered. The corrected section appears below:

Yifan Zhou^1, 2†^, Jin Wei^3, 4†^, Ning Wang^3, 4†^, Yisheng Chen^5†^, Cheng Fang^6^, Minwen Zhou^3, 4^, Xinrong Zhou^3, 4*^, Jianfeng Luo^6, 7, 8*^, Xiaodong Wang^9*^, Qing Peng^1*^

In the published article, there was an error in the **citation section**. The citation was incorrectly written as: Zhou Y, Wei J, Wang N, Chen Y, Fang C, Zhou M, Zhou X, Peng Q, Wang X and Luo J (2023) The prevalence, incidence, and persistence of self-reported visual impairment among Chinese population with diabetes mellitus: evaluation from a nationally representative survey, 2015–2018. *Front. Public Health*. 11:978457. doi: 10.3389/fpubh.2023.978457.

The corrected section appears below:

Zhou Y, Wei J, Wang N, Chen Y, Fang C, Zhou M, Zhou X, Luo J, Wang X and Peng Q (2023) The prevalence, incidence, and persistence of self-reported visual impairment among Chinese population with diabetes mellitus: evaluation from a nationally representative survey, 2015–2018. *Front. Public Health*. 11:978457. doi: 10.3389/fpubh.2023.978457.

In the published article, there was an error in the **copyright section**. The copyright section was incorrectly written as: © 2023 Zhou, Wei, Wang, Chen, Fang, Zhou, Zhou, Peng, Wang and Luo.

The corrected section appears below:

© 2023 Zhou, Wei, Wang, Chen, Fang, Zhou, Zhou, Luo, Wang, Peng

The authors apologize for these errors and state that this does not change the scientific conclusions of the article in any way. The original article has been updated.

